# Associated Factors of Dietary Patterns among Adolescents in the Rural Northern Region of Thailand: A Community-Based Cross-Sectional Study

**DOI:** 10.3390/healthcare12121215

**Published:** 2024-06-18

**Authors:** Penprapa Siviroj, Jukkrit Wungrath, Krongporn Ongprasert

**Affiliations:** 1 Department of Community Medicine, Faculty of Medicine, Chiang Mai University, Chiang Mai 50200, Thailand; penprapa.s@cmu.ac.th; 2 Faculty of Public Health, Chiang Mai University, Chiang Mai 50200, Thailand; jukkrit.w@cmu.ac.th

**Keywords:** adolescents, dietary diversity, health behaviors, nutrition, rural region

## Abstract

This cross-sectional study aims to explore the dietary patterns and associated factors of adolescents, which are often overlooked in nutrition data systems. Face-to-face interviews were conducted with 304 participants aged 10 to 19 in rural northern Thailand, utilizing both open recall and list-based 24 h recall techniques, with the data recorded online. Dietary diversity (DD) was assessed using ten food groups as per the Food and Agricultural Organization guidelines. We employed binary logistic regression and multivariable logistic regression analyses. Most participants consumed items from the grains, white roots, and tubers food group, while the nuts, seeds, and pulses food group was the least consumed. The mean number of food groups consumed was 5.23 ± 0.12, with no significant differences across gender and age groups, and participants reporting no influence of mass media on food choices were more likely to have inadequate DD (AOR = 2.94; 95% CI 1.38–6.28). Conversely, those not influenced by social media when choosing food (AOR = 0.45; 95% CI 0.21–0.96), who felt relaxed during meals (AOR = 0.33; 95% CI 0.19–0.59), and with no role in family meal decisions (AOR = 0.55; 95% CI 0.31–0.95) were less likely to have inadequate dietary diversity. We suggest that assisting adolescents with mealtime management and involving them in selecting healthy menus could improve their dietary variety. Moreover, future research should further investigate these mechanisms to inform strategies for improving DD in this age group.

## 1. Introduction

The World Health Organization (WHO) defines adolescents as individuals aged 10–19 [1]. Adolescence is a time of rapid growth, where nutrition significantly shapes physical development, reproductive health, and the risk of non-communicable diseases in adulthood [2]. Recognizing that no single food can provide all necessary nutrients for optimal health, dietary diversity (DD) is considered crucial for achieving micronutrient adequacy [3,4]. Recent studies focusing on adolescents in low- and middle-income countries have highlighted insufficient dietary intake from essential food sources such as meat, fruits, vegetables, milk products, and protein. Conversely, there is a concerning trend towards the consumption of nutrient-poor diets, including fast food, excessive snacking, and sugary treats [5,6,7,8]. The 2021 Thailand Global School-based Student Health Survey presented data from 5661 high school students highlighted similar issues. Among these students, 18.2% reported consuming fruit at least twice weekly, while 15.3% reported consuming vegetables at least three times weekly. However, a notably higher percentage, 39.3%, reported consuming fast food at least three times weekly. These findings underscore a concerning trend of inadequate fruit and vegetable intake among adolescents in Thailand [9].

The process of making food choices during adolescence differs significantly from that in both childhood and adulthood. In childhood, guardians usually guide food choices, whereas adults have more autonomy. Adolescents, however, are caught in between, with their decision-making process being more susceptible to environmental factors [10,11,12,13]. The impact of residential environments on dietary practices and diversity in infants and young children has been extensively studied, yielding varied results. A recent study in Thailand [14], drawing on secondary data from the 2019 Thailand Multiple Indicators Cluster Survey [15], found no notable correlation between living in rural or urban areas and DD. However, when considering the minimum acceptable diet, a composite measure encompassing both dietary variety and meal frequency [16], analysis across multiple countries in South Asia including Afghanistan, Bangladesh, India, Maldives, Nepal, and Pakistan revealed a higher percentage of urban children (15.6%) meeting the standard compared to their rural counterparts (11.8%) [17]. Conversely, a study from Nepal indicated that children in rural areas were 1.45 times more likely to achieve the minimum acceptable diet. These conflicting findings suggest that residential location may not be the sole factor influencing dietary practices or diversity among adolescents. Additionally, there is an absence of data on adolescents in rural areas of Thailand, necessitating further investigation into the factors impacting their dietary practices and diversity. Evidence suggests that several factors can influence food choices among adolescents. Household circumstances such as low economic status and living in larger families have consistently been linked to insufficient dietary variety [18]. Moreover, parental attributes and parenting approaches, such as family meal routines and setting examples of healthy eating, along with maternal occupation, can shape eating habits [19,20]. Furthermore, during adolescence, individuals gain more independence, which makes influences beyond their families more impactful compared to childhood. Prolonged exposure to social platforms and concerns about body image have been correlated with dietary diversity [20]. These influences can have both positive and negative effects on shaping lifelong eating habits.

Despite the crucial role of the dietary patterns and DD in shaping the health and progress of adolescents, there are limited data in global nutrition addressing this age group and a discrepancy exists between the abundant nutritional data for children under five and the scarce and inconsistent findings for adolescents, both globally and in Thailand [2]. Therefore, this study aims to investigate DD—which is defined as the number of food items or food groups consumed within a specified period [3]—among adolescents aged 10 to 19 in rural northern Thailand. By examining current eating practices and factors associated with DD, this research may provide crucial insights for the initial planning of health promotion strategies aimed at maintaining DD among adolescents and ensure positive health outcomes.

## 2. Materials and Methods

### 2.1. Study Setting 

Rural areas constitute 47.11 percent of Thailand’s population, surpassing the global average of 38.97 percent across 196 countries [21,22]. Geographically, Thailand is divided into four primary regions: north, central, northeast, and south. The north of Thailand is distinguished by its numerous mountain ranges. Chiang Mai, a major city in the north, consists of 25 districts. The focus of this study was on adolescents from Samoeng District, characterized by its rural, mountainous terrain, where the majority of inhabitants are Thai nationals who predominantly speak Thai and adhere to Buddhism [23]. All participants in this study are students from schools within the study area. These schools receive meal support facilitated by the School Health Program in Samoeng District, a governmental organization tasked with supporting funding and providing knowledge to improve student nutrition. The lunch menu each day follows a consistent pattern for all students, with most meals featuring rice as the main energy source. These are accompanied by two side dishes or a mixed dish, such as fried rice, fried noodles, or noodles with soup.

### 2.2. Study Design and Sample Size Calculation 

In this community-based cross-sectional study, we utilized secondary data from the School Health Program in Samoeng District to identify 1249 eligible children for participation. Our selection criteria for schools focused on choosing the top three schools with the highest number of students. We calculated the sample size using the Epi Info^TM^ version 5.5.15 [24] application to estimate the study population. For sample size calculation, we used a population (N) of 1249 children based on an expected frequency of 15.3%, from a previous study in rural Ghana [8], which revealed the proportion of adolescents with adequate DD. We took into account a 95% confidence level, a 5% acceptable margin of error, and the design effect of 2.0. Consequently, the sample comprised 344 individuals. 

### 2.3. Participants

Participants were recruited through study posters placed in classrooms and posted on the schools’ social media platforms. Interested parents and students were invited to attend group information sessions at the school, held on the date and time indicated on the poster. Following the group sessions, individual discussions were held for interested parents or students. Before providing information, students under 13 years of age provided assent forms, separate from the consent forms obtained from their parents. Students aged 13 to 17 provided informed consent forms co-signed by their parents, while students aged 18 to 19 provided consent forms independently. Initially, 350 students provided consent. However, 46 students were excluded or unable to complete the questionnaire, resulting in 304 students ultimately participating in our study. To be eligible, students had to be between 10 and 19 years old. The exclusion criteria were as follows: (a) individuals or their parents or guardians who were illiterate in Thai; (b) individuals who reported significant deviations from their typical school day meals within the 24 h preceding the interview, such as deviations due to illness or special occasions (e.g., feast days or wedding ceremonies); and (c) individuals who adhered to specific dietary restrictions influenced by cultural or religious beliefs, such as vegan or vegetarian diets.

### 2.4. Data Collection Procedure

Data collection occurred between 31 October 2023 and 28 February 2024. Interviews were scheduled from Tuesday to Friday to gather 24 h food records exclusively on school days. These interviews, conducted one-on-one, lasted approximately 20 min and utilized a web-based application. The interviewers were fourth-year medical students who were well-trained in using web-based applications for data collection, conducting interviews, and addressing ethical issues, and they were supervised by the principal investigator.

### 2.5. Operational Definition 

This study implemented the DD indicator outlined in the 2021 Food and Agricultural Organization (FAO) guidelines for Minimum Dietary Diversity for Women [3]. Each individual was assigned a score based on their consumption of food groups during the preceding day and night using the 24 h recall data collection method, with a score of 1 indicating consumption and 0 indicating non-consumption. Therefore, the dietary diversity score (DDS) ranged from 0 to 10 per participant, with a score of less than 5 indicating inadequate dietary diversity. Items consumed in amounts less than 15 g, approximately one tablespoon, were excluded from the count. Therefore, ingredients used in mixed dishes were not included in the analysis.

### 2.6. Survey Questionnaire

The questionnaire comprised four sections: (1) demographic data; (2) factors influencing food choices, as well as family eating habits; (3) food open recall on 24 h recall; and (4) food list based on 24 h recall (list of healthy and unhealthy foods).

(1) Demographic data on adolescent characteristics were collected, including information on age, gender, education level, daily pocket money, household composition (including who the participant lived with), the primary individual responsible for meal preparation (including their age, education level, and occupation), household size, and monthly household income.
(2) The factors influencing food choices, as well as family eating habits, were reviewed and adapted from previous studies [3,8,16,18,19,20,25]. We collected data on both internal and external motivations influencing food choices. Internal factors included personal preference, taste, appearance, nutrient components, and avoiding the risk of obesity. External factors included influences from family members, peers, social media, mass media, time restrictions, the placement of food, and financial considerations. Additionally, we examined family eating habits, which ranged from meal locations to involvement in meal preparation. These habits included eating at home; preparing meals in-house; having breakfast, lunch, and dinner with family members; and participating in menu selection and meal preparation. All factors influencing food choices were assessed using closed-ended (yes or no) questions.
(3) Food open recall on 24 h recall: In the open recall method, participants are asked to list the foods they consumed chronologically, starting with the first food eaten and ending with the last one at the end of the day or night. Participants can respond with single food items or mixed dishes. If they mention mixed dishes, the interviewer will inquire about the specific ingredients consumed. The interviewer will then record the food items under each relevant food group in the interface used for the list-based questionnaire.
(4) Food list based on 24 h recall: In this method, the interviewer reads a list of foods and beverages to the respondent. The enumerator informs respondents that they should respond yes for each food or beverage consumed during the specified 24 h recall period.

The healthy foods list in the initial draft of the list-based questionnaire underwent translation and grouping based on the DD indicator outlined in the 2021 FAO guidelines for Minimum Dietary Diversity for Women [3]. We used the Women of Reproductive Age (WRA) to categorize foods into ten groups as follows: (1) grains, white roots, and tubers; (2) pulses; (3) nuts and seeds; (4) milk and milk products; (5) meat, poultry, and fish; (6) eggs; (7) dark green leafy vegetables; (8) other vitamin A-rich fruits and vegetables; (9) other vegetables; and (10) other fruits. The unhealthy foods list was translated and adapted from the same source as the healthy foods list, with additional input from the Indicators for Assessing Infant and Young Child Feeding Practices, 2021 [16]. The regional nutrition specialist enhanced the first draft by integrating locally accessible foods to ensure their incorporation into each food group. Regarding unhealthy food groups, we divided them into nine categories using WRA: (1) sweet or flavored milk; (2) any type of fruit juice; (3) sweetened carbonated beverages; (4) other sweet beverages; (5) baked or fried sugary sweets; (6) sugary confectionery; (7) frozen desserts; (8) sentinel fried and salty foods; and (9) other unhealthy snacks. The final lists were curated to include commonly accessible foods in the study area. Specifically, the healthy food list comprises 107 items, while the unhealthy food list consists of 44 items.

The questionnaire underwent evaluation by specialists, including two pediatricians and one nutritionist, who collaborated to refine it. Each item in the questionnaire was deemed acceptable if it attained a Content Validity Index (CVI) score of at least 0.78 [26]. Following this, the questionnaire was administered to a similar population group for further evaluation. Specifically, it was given to twenty high school students from another school who were not included in this study, to assess the effectiveness of the language and flow of the interview process.

### 2.7. Statistical Analysis 

All statistical analyses were conducted using the STATA version 16 statistical software program (Stata Corp. 2019. Stata Statistical Software: Release 16, Stata Corp LLC, College Station, TX, USA). Continuous variables are presented as means ± standard deviation (SD), and categorical data are presented as frequencies and percentages. The chi-square test was utilized to examine the association of categorical variables between two groups of participants: the “Adequate DD Group”, consisting of individuals consuming five or more food groups out of ten healthy options, and the “Inadequate DD Group” consisting of individuals consuming fewer than five healthy food groups out of the total ten. Bivariate and multivariable logistic regression analyses were used to examine the association between motivation factors, family eating habits, and DD among adolescents, adjusting for education level and household size. The crude odds ratio (COR) and adjusted odds ratio (AOR) with 95% confidence intervals (CIs) were reported to represent the magnitude of the association. The results of this study were reported according to the Strengthening of the Reporting of Observational Studies in Epidemiology (STROBE) recommendations for cross-sectional studies [27]. All statistical analyses were two-sided, and a *p*-value of 0.05 was considered to be statistically significant. 

### 2.8. Ethical Considerations 

This study was conducted in accordance with the Declaration of Helsinki, and approved by the Ethics Committee of the Faculty of Medicine, Chiang Mai University, Thailand (protocol code: 458/2023 and date of approval: 18 October 2023).

## 3. Results

### 3.1. Characteristics of Study Participants 

One-third of the participants were categorized as having inadequate DD. Table 1 presents the characteristics of the study participants, comprising 304 students aged between 10 and 19 years old. The median age of the group was 14.0 years, with an interquartile range of 3.0 years. Most participants (85.5%) received less than USD 0.8 as daily pocket money, which is equivalent to the cost of one serving of street food. Mothers (69.1%) were primarily responsible for meal preparation, with the majority aged 36 to 55 (57.9%) and having completed high school education (47.1%), though nearly one-fifth lacked formal education. The majority of the individuals primarily responsible for preparing meals (55.9%) have jobs that allow for them to work from home with a flexible schedule. The monthly incomes of the participants’ families ranged from USD 135 to 270. 

### 3.2. Healthy Dietary Consumption and Dietary Diversity 

**Figure 1:**
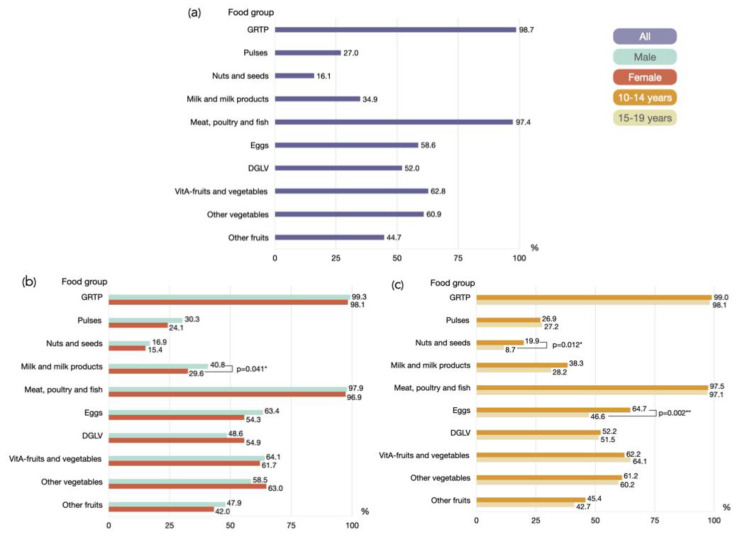
Histograms illustrate the proportion of healthy dietary consumption among adolescents by (**a**) all participants, (**b**) gender, and (**c**) age group during the previous day or night. * *p*-value < 0.05; ** *p*-value < 0.01; statistical analysis with Chi-square test. Abbreviations: GRTP: grains, white roots and tubers, and plantains; DGLV: dark green leafy vegetables; VitA-fruits and vegetables: other vitamin A-rich fruits and vegetables.

The proportions of participants consuming various food groups during the previous night are depicted in Figure 1. The majority of the participants consumed grains, white roots, and tubers (98.7%), followed closely by meat, poultry, and fish (97.4%). Other vitamin A-rich fruits and vegetables, other vegetables, and eggs were consumed by nearly two-thirds of the participants, with rates of 62.8%, 60.9%, and 58.6%, respectively. One-third of the participants consumed milk and milk products (34.9%). The lowest proportions of consumption were observed for pulses (27.0%), followed by nuts and seeds (16.1%). Males tend to consume a higher proportion of all food groups compared to females, with the exception of dark green leafy vegetables and other vegetables. Specifically, the consumption of milk and milk products is notably higher among males than females (Figure 1b). Additionally, the consumption of nuts and seeds, as well as eggs, is significantly higher among adolescents aged 10–14 compared to those aged 15–19 (Figure 1c).

**Figure 2:**
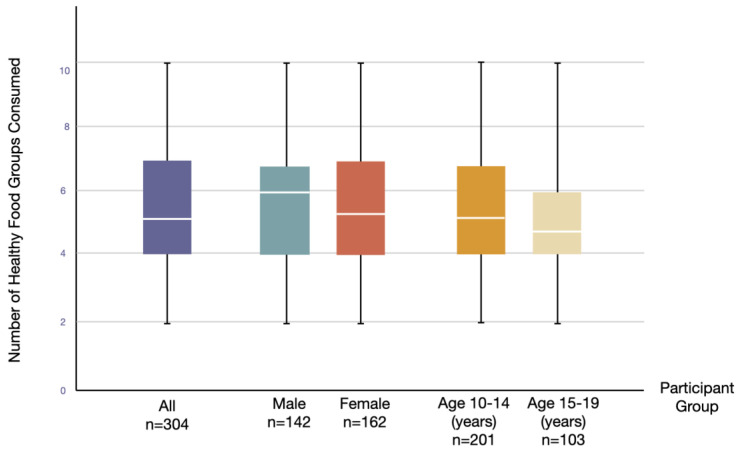
Box plots Illustrating the dietary diversity scores among all participants and classified by gender and age groups.

Additionally, the DDS of consumption is indicated in Figure 2. The mean and standard deviation (SD) of DDS for all participants was 5.23 ± 0.12. For males, the mean DDS was 5.67 ± 0.18, while for females, it was 5.40 ± 2.05. The mean score for participants aged 10–14 was 5.68 ± 2.27, and for those aged 15–19, it was 5.24 ± 1.92.

### 3.3. Unhealthy Food Consumption 

**Figure 3:**
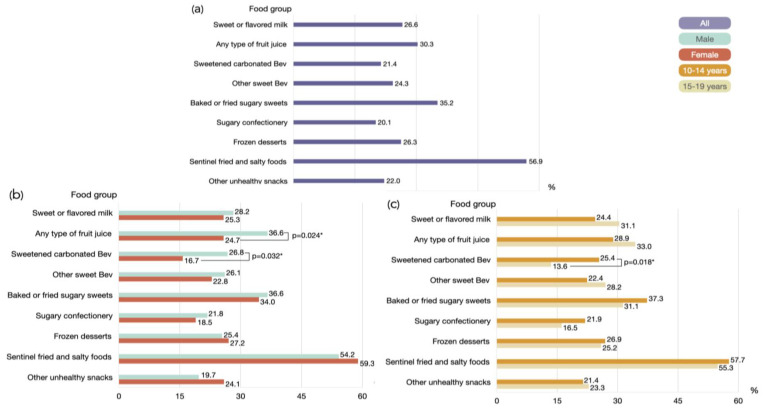
Histograms illustrate the proportion of unhealthy dietary consumption among adolescents by (**a**) all participants, (**b**) gender, and (**c**) age group during the previous day or night. * *p*-value < 0.05; statistical analysis with Chi-square test. Abbreviations: Bev: beverages.

As illustrated in Figure 3a, the highest proportion of unhealthy food groups during the previous day or night among the participants was sentinel fried and salty foods (56.9%), which include chips, crisps, cheese puffs, instant noodles, and similar items. Males tend to consume a higher proportion of unhealthy foods compared to females, with a few exceptions such as frozen desserts, fried and salty foods, and other unhealthy snacks. It highlights that there are significantly higher rates of consumption of fruit juice and sweetened carbonated beverages among males than females (Figure 3b). Furthermore, the result showed that the consumption of sweetened carbonated beverages is notably higher among adolescents aged 10–14 compared to those aged 15–19.

### 3.4. Factors Associated with Dietary Diversity 

The internal and external motivation factors that influenced food choices, as well as family eating habits, were analyzed using binary and multivariable logistic regression. Supplementary Tables S1–S3 provide descriptions of the factors influencing food choices, dietary consumption, and unhealthy consumption by food groups among adolescents. Table 2 presents data on the predictors of inadequate DD. It was found that students who reported that their choice of food selection was not influenced by mass media were significantly more likely to have inadequate DD, with a 2.94 times higher likelihood compared to those who were exposed to mass media content related to food choices (AOR = 2.94; 95% CI 1.38, 6.28, *p* = 0.005). In contrast, the participants who reported that their choice of food was not influenced by social media were 0.45 times less likely to have inadequate DD (AOR = 0.45; 95% CI 0.21, 0.96, *p* = 0.040) than those who were influenced by social media. Likewise, the participants who felt unrushed during mealtime were 0.33 times less likely to have inadequate DD compared to students who reported having time constraints during their meals (AOR = 0.33; 95% CI 0.19, 0.59, *p* ≤ 0.001). In addition, the participants who did not participate in selecting the menu for their family meals were 0.55 times less likely to have inadequate DD compared to those who were involved in the menu selection process (AOR = 0.55; 95% CI 0.31, 0.95, *p* = 0.033). 

## 4. Discussion

This study assessed the dietary patterns, DD, and associated factors of adolescents living in rural communities in the north of Thailand. Our findings indicate that the prevalence of inadequate DD among adolescents is 36.7%. This proportion is lower than those reported in recent studies conducted in Ethiopia in 2024 (62.2%) [18], Ghana in 2023 (84.6%) [8], Bangladesh in 2020 (42.3%) [28], and Uganda in 2020 (45.3%) (Supplementary Table S4). The differences in the prevalence of insufficient DD primarily stem from variations in study areas, which greatly influence outcomes. This variance persists even within the same country, as demonstrated by previous research indicating a significant range in prevalence, from 27% to 52% [29]. The variability in methods used to assess DD also contributes to the reported data discrepancies. Recent studies [8,18,28,30] have employed a 24 h recall of consumed foods, a method derived from the Food and Agriculture Organization of the United Nations (FAO). However, these assessment tools have different versions (2016 and 2021) and were adapted to local contexts, resulting in variations in each study’s protocol. 

Significant variations in the prevalence of inadequate DD are observed across different studies. In our study, the mean DDS was 5.5 ± 2.10, which only slightly surpasses the scores reported in recent studies [8,18,20,28,30]. This difference may be attributed to our study focusing only on school days; therefore, the management of school lunches can affect DD. Our study schools provided limited daily menus, which automatically guided the participants to choose from carefully planned meals emphasizing nutritional value. In contrast, in schools with broader cafeterias, adolescents had the opportunity to select from menus based on their personal preferences, which significantly influenced their food choices and potentially impacted their DD [25]. Grains emerged as the most consumed food type in our study, consistent with previous research [8,18,20,28,30]. The participants in our study exhibited a higher frequency of consuming fruits and vegetables compared to previous reports in Thailand’s national database [31]. This disparity might be attributed to socioeconomic differences in our study area, where agriculture prevails as the primary occupation and transportation to markets poses challenges due to mountainous terrain. Consequently, the local environment may foster higher consumption of fruits and vegetables, which are easy to cultivate in the region.

Our results show that different types of media have varying effects on behavioral outcomes. The participants who stated that mass media did not influence their food choices were nearly three times more likely to have inadequate DD compared to their counterparts. Conversely, those who reported that social media did not affect their food choices were less likely to have inadequate DD. These findings are consistent with recent studies suggesting that media exposure influences DD. A previous study found that adolescents who spent more time on social media were 2.6 times more likely to have low DD compared to those who spent less time on social media [20]. To gain a deeper understanding of these effects, it is necessary to further explore specific media-related factors that can influence adolescent behavior, such as the content viewed and the amount of time participants spend on both types of media [6,32]. In our study, students who did not feel rushed during meals were less likely to have inadequate DD compared to those who reported time constraints. This finding is consistent with prior research which reported that shorter meal durations can negatively influence adolescents’ food choices [25]. Limited time may result from longer wait times for food or a desire for more free time to play during breaks. Therefore, promoting longer meal durations and encouraging enjoyment without feeling rushed during meals could encourage adolescents to make more thoughtful food choices and opt for healthier options. Our study also revealed that students who did not participate in selecting family menus were less likely to have a balanced diet. This finding is consistent with prior research which demonstrated that parental feeding practices, such as controlling and restricting eating, were associated with better diet quality, whereas unconditional permission to eat was linked to poorer diet quality indices [33]. These effects can be attributed to the developmental stage of adolescents, where they begin to assert their autonomy but may lack sufficient health literacy and reasoning skills, leading to food choices based more on personal preference than nutritional value [25]. Therefore, during the transition from childhood to adulthood, adolescents should be guided toward healthier food choices, including involvement in family meal preparation, which has been shown to improve dietary quality and eating habits [34].

Our findings indicate that the types of media adolescents engage with have differing impacts on eating diversity. This underscores the importance of future studies focusing on amplifying positive influences and minimizing negative ones. Additionally, healthcare professionals should emphasize the importance of having adequate mealtimes and assist adolescents in selecting healthy family menus, which could potentially improve DD.

Our study has several limitations. Firstly, this study employed the recall method to explore the food consumed in the days preceding the interview, which can introduce recall bias and may provide an incomplete depiction of complete diets. To mitigate these limitations, the participants were given an exact appointment interview day and reminded to focus on their diet prior to this day. Secondly, our food list generated from the 24 h recall method has limitations in capturing all types of foods and beverages, such as water or unsweetened tea. Finally, the recruitment of exclusively adolescents from a single region limits the generalizability of our findings to broader cultural contexts. 

## 5. Conclusions

In this study, it was found that one-third of participants had inadequate DD. Our findings indicate that, in adolescents, feeling unrushed during mealtimes and not having a role in choosing the family menu were associated with lower rates of inadequate DD. Thus, assisting adolescents in managing their time effectively during meals and involving them in selecting family meal menus could potentially improve DD. Additionally, our results highlight that different types of media consumption have varying impacts on DD. Therefore, further exploration of the mechanisms underlying this influence could inform strategies aiming to maximize positive impacts and reduce negative ones.

## Figures and Tables

**Table 1:** Association between characteristics, household details, and dietary diversity (*n* = 304).

Variables	Frequency (*n*)	Percentage (%)	Dietary Diversity, *n* (%)		Chi-Square (χ^2^)	*p*-Value
			Inadequate (<5 Foods)	Adequate (≥5 Foods)		
Adolescent characteristics						
Age (years), median [IQR]	14.0 [3.0]					
10–14	201	66.1	75 (37.3)	126 (62.7)	0.009	0.925
15–19	103	33.9	39 (37.9)	64 (62.1)		
Gender (n%)						
Male	142	46.7	52 (36.6)	90 (63.4)	0.088	0.767
Female	162	53.3	62 (38.3)	100 (61.7)		
Education levels						
Grades 5 to 6	41	13.5	20 (48.8)	21 (51.2)	2.677	0.262
Grades 7 to 9	229	75.3	81 (35.4)	148 (64.6)		
Grades 10 to 12	34	11.2	13 (38.2)	21 (61.8)		
Pocket money per day (USD ^¥^), median [IQR]	20.0 [0.0]					
≤0.8	260	85.5	101 (38.8)	159 (61.2)	1.389	0.239
>0.8	44	14.5	13 (29.5)	31 (70.5)		
Household details						
Living with whom						
Both parents	222	73.0	84 (37.8)	138 (62.2)	0.797	0.671
Single parents	44	14.5	18 (40.9)	26 (68.4)		
Guardian	38	12.5	12 (31.6)	26 (68.4)		
Primary individual responsible for preparing meals						
Myself	23	7.6	10 (43.5)	13 (56.5)	3.892	0.273
Father	32	10.5	15 (46.9)	17 (53.1)		
Mother	210	69.1	79 (37.6)	131 (62.4)		
Grandparents/others	39	12.8	10 (25.6)	29 (74.4)		
Age of primary individual responsible for preparing meals (years)						
Median [IQR]	40.0 [10.0]					
<20	22	7.2	10 (45.5)	12 (54.5)	3.069	0.381
21–35	79	26.0	25 (31.6)	54 (68.4)		
36–55	176	57.9	71 (40.3)	105 (59.7)		
>55	27	8.9	8 (29.6)	19 (70.4)		
Education level of the primary individual responsible for preparing meals (*n* = 257)						
No education	46	17.9	20 (43.5)	26 (56.5)	3.946	0.267
Primary school (1–6 years)	66	25.7	22 (33.3)	44 (66.7)		
High school (7–12 years)	121	47.1	44 (36.4)	77 (63.6)		
Higher than high school (>12 years)	24	9.3	13 (54.2)	11 (45.8)		
Occupation of the primary individual responsible for preparing meals (*n* = 279)						
Stay-at-home guardian	27	9.7	11 (40.7)	16 (59.3)	4.181	0.243
Full-time work with day shifts	86	30.8	35 (40.7)	51 (59.3)		
Work with rotational shifts	10	3.6	6 (60.0)	4 (40.0)		
Work-from-home job with a flexible schedule	156	55.9	51 (32.7)	105 (67.3)		
Household size						
Up to 2 people	13	4.3	3 (23.1)	10 (76.9)	3.505	0.173
3–4 people	178	58.6	74 (41.6)	104 (58.4)		
≥5 people	112		37 (32.7)	76 (67.3)		
Monthly household income (USD ^¥^) (*n* = 130)						
<135	36	27.7	14 (38.9)	22 (61.1)	0.516	0.915
135 to 270	51	39.2	18 (35.3)	33 (64.7)		
270 to 405	25	19.2	10 (40.0)	15 (60.0)		
>405	18	13.8	8 (44.4)	10 (55.6)		

Ten food categories are considered recommendations by 2021 FAO guidelines. Individual dietary diversity is categorized as “inadequate dietary diversity” if <5 food groups are consumed and “adequate dietary diversity” if ≥5 food groups are consumed. Abbreviations: IQR: interquartile range; USD: United States dollars. ^¥^ = 1.00 USD = 36.63 Thai baht (2 April 2024).

**Table 2:** The associations between motivation factors, family eating habits, and dietary diversity (*n* = 304).

Variables	Category	Minimal Dietary Diversity (*n* = 304)		Crude OR ^a^ 95% CI	*p*-Value	Adjusted OR ^b^, 95% CI	*p*-Value
		<5 Foods	≥5 Foods				
Internal motivation factors							
Personal preference	No	39 (36.4)	68 (63.6)	0.93, 0.57 to 1.52	0.780	1.06, 0.55 to 2.02	0.867
	Yes	75 (38.1)	122 (61.9)	1		1	
AATaste	No	41 (38.3)	66 (61.7)	1.05, 0.65 to 1.71	0.828	0.91, 0.47 to 1.76	0.792
	Yes	73 (37.1)	124 (62.9)	1		1	
Appearance	No	80 (41.9)	111 (58.1)	1.67, 1.02 to 2.74	0.041	1.63, 0.88 to 3.03	0.120
	Yes	34 (30.1)	79 (69.9)	1		1	
Nutrient components	No	72 (41.9)	100 (58.1)	1.54, 0.96 to 2.48	0.074	1.73, 0.98 to 3.06	0.059
	Yes	42 (31.8)	90 (68.2)	1		1	
Not increasing the risk of obesity	No	89 (39.7)	135 (60.3)	1.45, 0.84 to 2.50	0.180	1.15, 0.62 to 2.16	0.656
	Yes	25 (31.3)	55 (68.8)	1		1	
External motivation factors							
Family member influence	No	49 (32.7)	101 (67.3)	0.66, 0.42 to 1.06	0.086	0.63, 0.36 to 1.09	0.097
	Yes	65 (42.2)	89 (57.8)	1		1	
Peer influence	No	79 (38.7)	125 (61.3)	1.17, 0.71 to 1.93	0.529	1.08, 0.59 to 1.97	0.814
	Yes	35 (35.0)	65 (65.0)	1		1	
Social media influence	No	78 (38.0)	127 (62.0)	1.07, 0.65 to 1.77	0.776	0.45, 0.21 to 0.96	0.040 *
	Yes	36 (36.4)	63 (63.6)	1		1	
Mass media influence	No	89 (42.2)	122 (57.8)	1.98, 1.16 to 3.38	0.012 *	2.94, 1.38 to 6.28	0.005 **
	Yes	25 (26.9)	68 (73.1)	1		1	
Time restrictions	No	50 (29.4)	120 (70.6)	0.46, 0.28 to 0.73	0.001 ***	0.33, 0.19 to 0.59	<0.001 ***
	Yes	64 (47.8)	70 (52.2)	1		1	
Placement of food influence	No	48 (34.8)	90 (65.2)	0.81, 0.51 to 1.29	0.373	0.97, 0.56 to 1.67	0.903
	Yes	66 (39.8)	100 (60.2)	1		1	
Financial motivations	No	45 (43.7)	58 (56.3)	1.48, 0.91 to 2.41	0.111	1.69, 0.97 to 2.94	0.065
	Yes	69 (34.3)	132 (65.7)	1		1	
Family’s eating habits							
Typically eating at home	No	10 (27.0)	27 (73.0)	0.58, 0.27 to 1.25	0.164	0.98, 0.34 to 2.87	0.976
	Yes	104 (39.0)	163 (61.0)	1		1	
Usually having meals that are prepared in-house	No	11 (29.7)	26 (70.3)	0.67, 0.32 to 1.42	0.300	0.73, 0.26 to 2.10	0.564
	Yes	103 (38.6)	164 (61.4)	1		1	
Typically having breakfast with other family members	No	43 (41.0)	62 (59.0)	1.25, 0.77 to 2.03	0.367	1.59, 0.84 to 3.00	0.153
	Yes	71 (35.7)	128 (64.3)	1		1	
Typically having lunch with other family members at the weekend	No	39 (35.5)	71 (64.5)	0.87, 0.54 to 1.42	0.579	1.02, 0.54 to 1.91	0.955
	Yes	75 (38.7)	119 (61.3)	1		1	
Typically having dinner with other family members	No	17 (29.8)	40 (70.2)	0.66, 0.35 to 1.22	0.186	0.52, 0.21 to 1.27	0.149
	Yes	97 (39.3)	150 (60.7)	1		1	
Playing a role in choosing the menu	No	57 (33.1)	115 (66.9)	0.65, 0.41 to 1.04	0.074	0.55, 0.31 to 0.95	0.033 *
	Yes	57 (43.2)	75 (56.8)	1		1	
Participate in preparing most of the meals in-home	No	41 (38.7)	65 (61.3)	1.08, 0.66 to 1.76	0.756	1.67, 0.92 to 3.05	0.091
	Yes	73 (36.9)	125 (63.1)	1		1	

Statistical analysis by ^a^ binary logistic regression and ^b^ multivariable logistic regression adjusted for education level and household size. * Significant association at *p* < 0.05, ** Significant association at *p* < 0.01, *** Significant association at *p* < 0.001. The reference group is adequate dietary diversity (≥5 food group). Abbreviations: CI: confidence interval; OR: odds ratio.

## Data Availability

The data presented in this study are available on request from the corresponding author on reasonable request.

## Supplementary Materials

The following supporting information can be downloaded at: https://www.mdpi.com/article/10.3390/healthcare12121215/s1, Table S1: Frequency and percentage of factors influencing food choices among adolescents; Table S2: Description of dietary consumption classified by food groups by 2021 FAO guidelines, during the previous day or night among adolescents with adequate and inadequate minimum dietary diversity; Table S3: Description of unhealthy consumption classified by food groups by 2021 FAO guidelines, during the previous day or night among adolescents with adequate and inadequate minimum dietary diversity; Table S4: Comparison between the most consumed food type in this study and previous studies.
