# Supplementary material for: Associated Factors of Dietary Patterns among Adolescents in the Rural Northern Region of Thailand: A Community-Based Cross-Sectional Study
Source: Healthcare (Basel). 2024 Jun 18;12(12):1215. doi: 10.3390/healthcare12121215 (PMC11203095; doi:10.3390/healthcare12121215)
Supplement: Supplementary file 1 [file healthcare-12-01215-s001.zip › Supplementary Table S1_Diet Diversity_180624.pdf]

Table S1. Frequency and percentage of factors influencing food choices among adolescents.

Variables	Influencing food choices (n = 304)	
	**No, n (%)**	**Yes, n (%)**
**Internal motivation factors**		
Personal preference	107 (35.2)	197 (64.8)
Taste	107 (35.2)	197 (64.8)
Appearance	191 (62.8)	113 (37.2)
Nutrient components	172 (56.6)	132 (43.4)
Not increasing the risk of obesity	224 (73.7)	80 (26.3)
**External motivation factors**		
Family member influence	150 (49.3)	154 (50.7)
Peer influence	204 (67.1)	100 (32.9)
Social media influence	205 (67.4)	99 (32.6)
Mass media influence	211 (69.4)	93 (30.6)
Time restrictions	170 (55.9)	134 (44.1)
Placement of food influence	138 (45.4)	166 (54.6)
Financial motivations	103 (33.9)	201 (66.1)
**Family's eating habits**		
Typically eating at home	37 (12.2)	267 (87.8)
Usually having meals that are prepared in-house	37 (12.2)	267 (87.7)
Typically having breakfast with other family members	105 (34.5)	199 (65.5)
Typically having lunch with other family members at the weekend	110 (36.2)	194 (62.8)
Typically having dinner with other family members	57 (18.8)	247 ((81.3)
Playing a role in choosing the menu	172 (56.6)	132 (43.4)
Participate in preparing most of the meals in-home	106 (34.6)	198 (65.1)
