# Supplementary material for: Associated Factors of Dietary Patterns among Adolescents in the Rural Northern Region of Thailand: A Community-Based Cross-Sectional Study
Source: Healthcare (Basel). 2024 Jun 18;12(12):1215. doi: 10.3390/healthcare12121215 (PMC11203095; doi:10.3390/healthcare12121215)
Supplement: Supplementary file 1 [file healthcare-12-01215-s001.zip › Supplementary Table S2_Diet Diversity_180624.pdf]

Table S2. Description of dietary consumption classified by food groups by WRA, during the previous day or night among adolescents with adequate and inadequate minimum dietary diversity.

**Consuming food groups**	**n (%)**			***p*-value**
	**Total**	**Minimum dietary diversity**		
		**< 5 foods**	**≥ 5 foods**	
n	304	114	190	
Grain, white roots, and tubers	300 (98.7)	110 (96.5)	190 (100.0)	0.019 [a,**]
Pulses	82 (27.0)	5 (4.4)	77 (40.5)	< 0.001 [b,***]
Nuts and seeds	49 (16.1)	1 (0.9)	48 (25.3)	< 0.001 [b,***]
Milk and milk products	106 (34.9)	10 (8.8)	96 (50.5%)	< 0.001 [b,***]
Meat, poultry, and fish	296 (97.4)	109 (95.6)	187 (98.4)	0.134 [a]
Egg	178 (58.6)	40 (35.1)	138 (72.6)	< 0.001 [b,***]
Dark green leafy vegetables	158 (52.0)	23 (20.2)	135 (71.1)	< 0.001 [b,***]
Other vitamin A-rich fruits and vegetables	191 (62.8)	38 (33.3)	153 (80.5)	< 0.001 [b,***]
Other vegetables	185 (60.9)	28 (24.6)	157 (82.6)	< 0.001 [b,***]
Other fruits	136 (44.7)	14 (12.3)	122 (64.2)	< 0.001 [b,***]

Significant p-values were analyzed using [a] Fisher's exact test, b chi-square test; *Significant association at p<0.05, ** Significant association at p <0.01; *** Significant association at p <0.001; WRA, Women of reproductive age.
