# Supplementary material for: Associated Factors of Dietary Patterns among Adolescents in the Rural Northern Region of Thailand: A Community-Based Cross-Sectional Study
Source: Healthcare (Basel). 2024 Jun 18;12(12):1215. doi: 10.3390/healthcare12121215 (PMC11203095; doi:10.3390/healthcare12121215)
Supplement: Supplementary file 1 [file healthcare-12-01215-s001.zip › Supplementary Table S3_Diet Diversity_180624.pdf]

Table S3. Description of unhealthy consumption classified by food groups by WRA, during the previous day or night among adolescents with adequate and inadequate minimum dietary diversity.

**Consuming unhealthy food groups**	**n (%)** **Total**	**Minimum dietary diversity**		***p*-value**
		**< 5 foods**	**≥ 5 foods**	
n	304	114	190	
Sweet or flavoured type of milk	81 (26.6)	9 (7.9)	72 (37.9)	< 0.001 [a],***
Any type of fruit juice	92 (30.3)	21 (18.4)	71 (37.4)	0.001 [a],***
Sweetened carbonated beverages	65 (21.4)	20 (17.5)	45 (23.7)	0.248
Other sweet beverages	74 (24.3)	27 (23.7)	47 (24.7)	0.891
Baked or fried sugary sweets	107 (35.2)	22 (19.3)	85 (44.7)	< 0.001 [a],***
Sugary confectionery	61 (20.1)	11 (9.6)	50 (26.3)	0.001 [a],***
Frozen desserts	80 (26.3)	18 (15.8)	62 (32.6)	0.002 **
Sentinel fried and salty foods	173 (56.9)	58 (50.9)	115 (60.5)	0.120
Other unhealthy snacks	67 (22.0)	23 (20.2)	44 (23.2)	0.571

Significant p-values were analyzed using [a] Fisher's exact test, b chi-square test; *Significant association at $p<0.05$, ** Significant association at $p <0.01$; *** Significant association at $p < 0.001$; WRA, Women of reproductive age.
