# Supplementary material for: Associated Factors of Dietary Patterns among Adolescents in the Rural Northern Region of Thailand: A Community-Based Cross-Sectional Study
Source: Healthcare (Basel). 2024 Jun 18;12(12):1215. doi: 10.3390/healthcare12121215 (PMC11203095; doi:10.3390/healthcare12121215)
Supplement: Supplementary file 1 [file healthcare-12-01215-s001.zip › Supplementary Table S4_Diet Diversity_180624.pdf]

Supplementary Table S4. Comparison between the most consumed food type in this study and previous studies.

Year Author /country	Age /Sample size	Inade-quade DD (%)	Mean ±SD DDS	Grains (%)	Pulses (%)	Nuts, seeds (%)	Milk and products (%)	Meat, poultry, fish	Eggs (%)	DGLV (%)	VAFV (%)	Other vege-tables (%)	Other fruits (%)
2024 Kera Ethiopia[a] [22]	17-19 (girl)/ 374	62.6	4.37 ±1.38	100	90.4	20.60	42.2	18.4	28.3	33.7	35.8	69.8	44.4
2023 Wiafe /Ghana[b] [8]	10-14 /137	84.7	3.8 ±0.8	100	10.2	34.1	8	86.9	7.3	29.9	2.2	81	7.3
2022 Worku Ethiopia[C] [17]	14-16 (girl) /292	43.3	4.9 ±1.4	83.5	N/A	80.6[1]	44	41.2	41.2	51.8	80.6[2]	47.9[3]	
2020 Islam Bangladesh[b] [29]	N/A /2463	42.3	4.84	99.9	46.2[6]		30.5	35.2[7] /73.2[8]	34.9,	26.8	31.4	60.7	45.1
2020 Isabirye Uganda[d] [20]	10-19 /598	45.3	N/A	99.7	66.2[8]		32.9	33.1	11.2	42.3	33.7	53.8	8.2

[a] Dietary diversity score was adopted from Minimum dietary diversity for women: A guide for measurement; by FAO 2021. A 24-hour recall was used to collect data. Inadequate dietary diversity: consume less than five food groups out of ten food groups.

[b] Dietary diversity score was adopted from Minimum dietary diversity for women: A guide for measurement; by FAO 2016. A 24-hour recall was used to collect data. Inadequate dietary diversity: consume less than five food groups out of ten food groups.

[c] Dietary diversity score was adopted from the Food and Nutrition Technical Assistance; Minimum dietary diversity for women: a guide for measurement, 2016. A 24-hour recall was used to collect data Inadequate (low) dietary diversity: consume less than five food groups out of nine food groups.

[d] Dietary diversity score was adopted from the Food and Nutrition Technical Assistance; Minimum dietary diversity for women: a guide for measurement, 2016. A 24-hour recall was used to collect data Inadequate (low) dietary diversity: consume less than four food groups out of nine food groups.

[1] Legumes; [2] vitamin A-rich fruits and vegetables; [3] Other fruits and vegetables; [4] Other and vitamin-A rich fruits; [5] Other and vitamin-A rich vegetables; [6] Legumes, nuts, and seeds; [7] Flesh and organ meat; [8] Pulses, nuts and seeds.

Abbreviations: DD, dietary diversity; DDS, dietary diversity score; DGLV, Dark green leafy vegetable; VAFV, vitamin A-rich fruits and vegetables.
